# Identification of *Vibrio ponticus* as a bacterial pathogen of coral trout *Plectropomus leopardus*

**DOI:** 10.3389/fcimb.2022.1089247

**Published:** 2022-12-23

**Authors:** Chunlei Gai, Jie Liu, Xurui Zheng, La Xu, Haibin Ye

**Affiliations:** ^1^ Marine Science Research Institute of Shandong Province, Qingdao, Shandong, China; ^2^ National Pathogen Collection Center for Aquatic Animals, Shanghai Ocean University, Shanghai, China; ^3^ Key Laboratory of Freshwater Fishery Germplasm Resources, Ministry of Agriculture and Rural Affairs of China, Shanghai, China

**Keywords:** *Vibrio ponticus*, *Plectropomus leopardus*, histopathological characterization, virulence, antibiotic susceptibility

## Abstract

*Vibrio ponticus* is a vital pathogen with potential danger for aquaculture animals. Yet *V. ponticus* pathogenic to the coral trout *Plectropomus leopardus* is still unknown. In this study, a virulent bacterial strain, temporarily named DX2, was isolated from diseased coral trout suffering liver necrosis with cell vacuolar degeneration, and was identified molecularly and phenotypically as *V. ponticus*. Besides, the DX2 isolate showed an LD_50_ value of 6.64×10^5^ CFU mL^-1^, developed multiple resistances to cephalosporins, macrolides, penicillins, peptides, and sulfonamides antimicrobials, and was highly susceptible to doxycycline and florfenicol in aquaculture use. To the best of our knowledge, this is the first report of the pathogenicity of *V. ponticus* to the coral trout, and the findings provide a reference for the control of pathogenic *V. ponticus* in the coral trout.

## Introduction

The coral trout *Plectropomus leopardus* is a commercially important tropical marine fish and widely distributed from the Western Pacific to East Africa and the Red Sea (Yoseda et al., 2008). Nowadays, the coral trout has been widely farmed in China along the southern coast in the tropical and subtropical regions through the implementation of modern farming techniques (Ma et al., 2015). However, this aquacultural industry has been badly affected by bacterial diseases such as tail fester disease and nodular disease under intensive culture conditions (Gu et al., 2015; Xu et al., 2019).

*Vibrio ponticus* is considered as an aquaculture pathogen that is widely distributed among aquatic environments (Macián et al., 2004). To date, diseases caused by *V. ponticus* have resulted in mass mortalities in Japanese sea bass *Lateolabrax japonicus* (Xie et al., 2007), maroon clownfish *Premnas biaculeatus* (Kim et al., 2007), cobia *Rachycentron canadus* (Sharma and Dube, 2017), golden pompano *Trachinotus ovatus* (Liu et al., 2018), and large yellow croaker *Larimichthys crocea* (You, 2018). Yet scarce information is available on *V. ponticus* as a causative pathogen of the coral trout.

In this study, a pathogenic strain of *V. ponticus* (DX2) was demonstrated as a pathogen of diseased coral trout with a typical symptom of liver necrosis, and its taxonomic position, virulence, as well as susceptibility to antibiotics were examined. As far as we know, this study is the first to identify *V. ponticus* as a causative pathogen of the coral trout. The findings of this study provide a reference for the control of pathogenic *V. ponticus* in the coral trout.

## Materials and methods

### Experimental fish

Twenty diseased coral trout (0.5-year-old, 70.42±15.50 g in weight), which were reared in concrete tanks, were obtained from an infected fish farm in Wenchang, Hainan, China in March 2022, and immediately placed into ice-cold sterile bags and sent to the laboratory according to Hossain et al. (2011). The water quality indicators measured during the disease outbreak were 28 °C, pH 8.0, salinity of 24, 7.8 mg L^-1^ dissolved oxygen, 0.20 mg L^-1^ nitrite, and 0.68 mg L^-1^ total ammonia. Healthy coral trout (31.04±5.17 g) were purchased from a fish farm in Changjiang, Hainan, China, and maintained good health with no contamination with *L. anguillarum*, *P. damsela, V. harveyi* pathogens by sampling and assessment according to Xu et al. (2008); Sun et al. (2015), and Carraro et al. (2018).

### Confirmation of causative pathogen

The potential pathogens were examined according to the previously described method (Wang et al., 2020). Firstly, the organs (liver, kidney, intestine, muscle, and gill) were dissected from the diseased coral trout in the laboratory, compressed manually between two glass slides to prepare the thin sections of organs, and then subjected to a observation for potential parasites using microscopy (Yang, 2018). Secondly, to determine whether this disease was caused by virus, bacteria-free organ filtrates were prepared according to Gong et al. (2010), and two replicate aquaria of healthy coral trout (10 fish per aquarium) were artificially infected with 0.2 mL of the freshly-prepared bacteria-free organ filtrates by intramuscular injection. Another two replicate aquaria of control coral trout (10 fish per aquarium) were treated intramuscularly with the same volume (0.2 mL) of sterile normal saline. All the test fish were stocked in aquaria containing 100 L natural aerated seawater at 28 °C and observed for fifteen days to record fish mortalities and any pathological signs. Thirdly, in order to determine if this disease was due to bacterial infection, liver samples were streaked onto thiosulfate citrate bile salt sucrose (TCBS) agar plate (Sinopharm Chemical Reagent Co., Ltd) for bacterial isolation according to Gu et al. (2015), and incubated at 28°C for 24h, then the uniform isolates were subjected to purification by repeated streaking onto nutrient agar (NA) plates amended with 15% NaCl. After the observation of the isolates’ colony and cell morphologies for purity assessment, the suspensions of pure isolates were further prepared by washing the inoculated NA plates after 24 h of incubation at 28 °C, and the colony forming units (CFU) in the suspensions were further estimated by calculating CFU on NA plates from a series of 10-fold dilutions in sterile normal saline. Afterwards, two replicate aquaria of healthy coral trout (10 fish per aquarium) were artificially infected by intramuscular injection with 0.2 mL of each pure isolate with 3.0 × 10^7^ CFU mL^-1^ (Yao et al., 2015). Another two replicate aquaria of control coral trout (10 fish per aquarium) were treated with the same volume (0.2 mL) of sterile normal saline intramuscularly. All the test fish were stocked in aquaria containing 100 L natural aerated seawater at 28 °C and observed carefully for seven days to record fish mortalities and any pathological signs. The challenge isolate was re-isolated from freshly dead fish to confirm the cause of death, and histopathological changes in the liver of infected and healthy fish were also examined as described by Phrompanya et al. (2021).

### Identification of causative pathogen

The identification of the pathogenic isolate was performed using 16S rRNA gene sequencing analysis and biochemical tests (Liu et al., 2018). The total DNA was extracted from the pathogenic isolate by the TIANamp DNA Kit (Tiangen Biotech. Co., Ltd., Beijing, China) following the manufacturer’s instructions. Then, the amplification of the 16S rRNA gene was conducted as described by Liu et al. (2018) using the universal primer pair 27F (5’-AGAGTTTGATCCTGGCTCAG-3’) and 1492R (5’-GGTTACCTTGTTACGACTT-3’), and the amplified product was subjected to sequencing through the ABI 3730 XL DNA Sequencer (Applied Biosystems, Waltham, MA, USA). Finally, the 16S rRNA gene sequence of the pathogenic isolate was subjected to the Basic Local Alignment Search Tool (BLAST) to search the closest related sequences in GenBank, and the construction of a phylogenetic tree was carried out using the neighbor-joining (NJ) method. Besides, the pathogenic isolate was phenotypically identified using API 20E identification kit (Biomerieux, France) following the guidance of manufacturer (Xie et al., 2007). The phenotypic features of *V. ponticus* previously described by Kim et al. (2007); Liu et al. (2018) and You (2018) were used as controls.

### Virulence assay

The virulence of the pathogenic isolate was examined by testing the mean lethal dose (LD_50_) in the coral trout according to Gu et al. (2015). Three replicate aquaria of healthy coral trout (10 fish per aquarium) were artificially infected by intramuscular injection with 0.2 mL of the pathogenic isolate with 2.1 × 10^4^, 2.1 × 10^5^, 2.1 × 10^6^, and 2.1 × 10^7^ CFU mL^-1^. Another three replicate aquaria of control coral trout (10 fish per aquarium) were treated intramuscularly with the same volume (0.2 mL) of sterile normal saline. All the test fish were stocked in aquaria containing 100 L aerated seawater at 28 °C without water being changed and observed carefully for seven days to record fish mortalities and any pathological signs. The challenge isolate was re-isolated from freshly dead fish to confirm the cause of death. Based on the logarithms of challenge doses and cumulative mortalities of challenged fish, the medium lethal dose (LD_50_) value was estimated using the Kochi method (Li et al., 2012) to evaluate the virulence of the pathogenic isolate.

### Susceptibility to antibiotics assay

The susceptibility of the pathogenic isolate to amoxicillin, ampicillin, azithromycin, bacitracin, cefotaxime, cefradine, ceftizoxime, cotrimoxazole, chloramphenicol, doxycycline, enoxacin, erythromycin, florfenicol, gentamycin, kanamycin, kitasamycin, netilmicin, nalidixic acid, novobiocin, oxacillin, penicillin, pipemidic acid, polymyxin B, rifampicin, roxithromycinum, and tobramycin was tested in triplicate by the Kirby-Bauer disk diffusion method (Joseph et al., 2011). Briefly, the pathogenic isolate was spread onto NA plates amended with 15% NaCl and antibiotic discs were then immediately placed on the inoculated plates. Afterward, the plates with antibiotic discs were incubated at 28 °C for 24 h to measure the diameters of inhibition zones surrounding the antibiotic discs. The susceptibility of the pathogenic isolate to twenty-six antimicrobials was evaluated by the instruction of the manufacturer (Hangzhou Binhe Microorganism Reagent Co., Ltd., Hangzhou, China). The antibiotic susceptibilities of *V. ponticus* previously reported by Kim et al. (2007); You (2018); Liu et al. (2018) and Kumari et al. (2020) were used as references.

## Results

### Confirmation of causative pathogen

**Figure 1:**
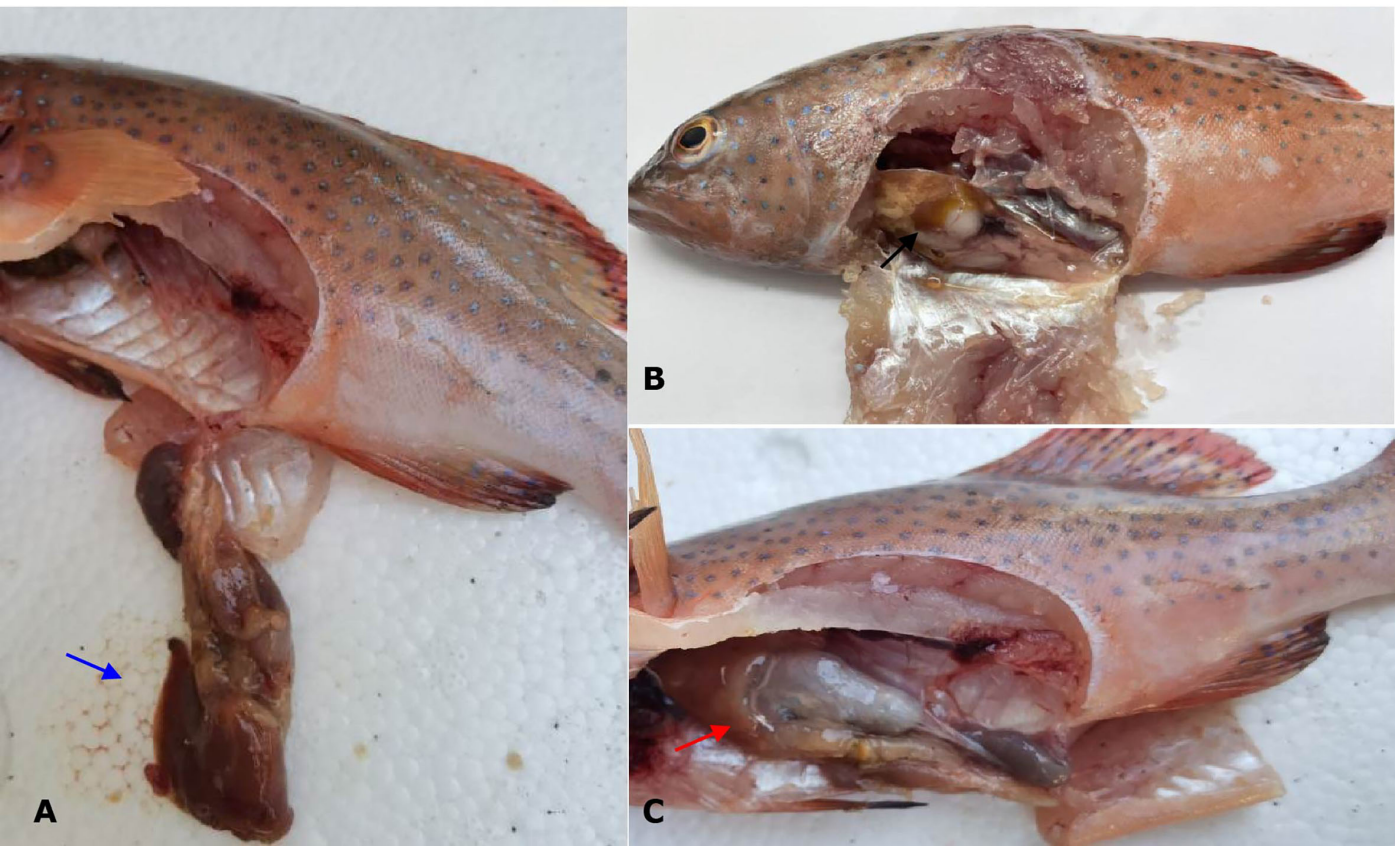
Gross signs of diseased coral trout. **(A)** Healthy fish. Blue arrow shows normal liver. **(B)** Naturally infected fish. Black arrow shows necrotic liver. **(C)** Experimental infected fish. Red arrow shows necrotic liver.

**Figure 2:**
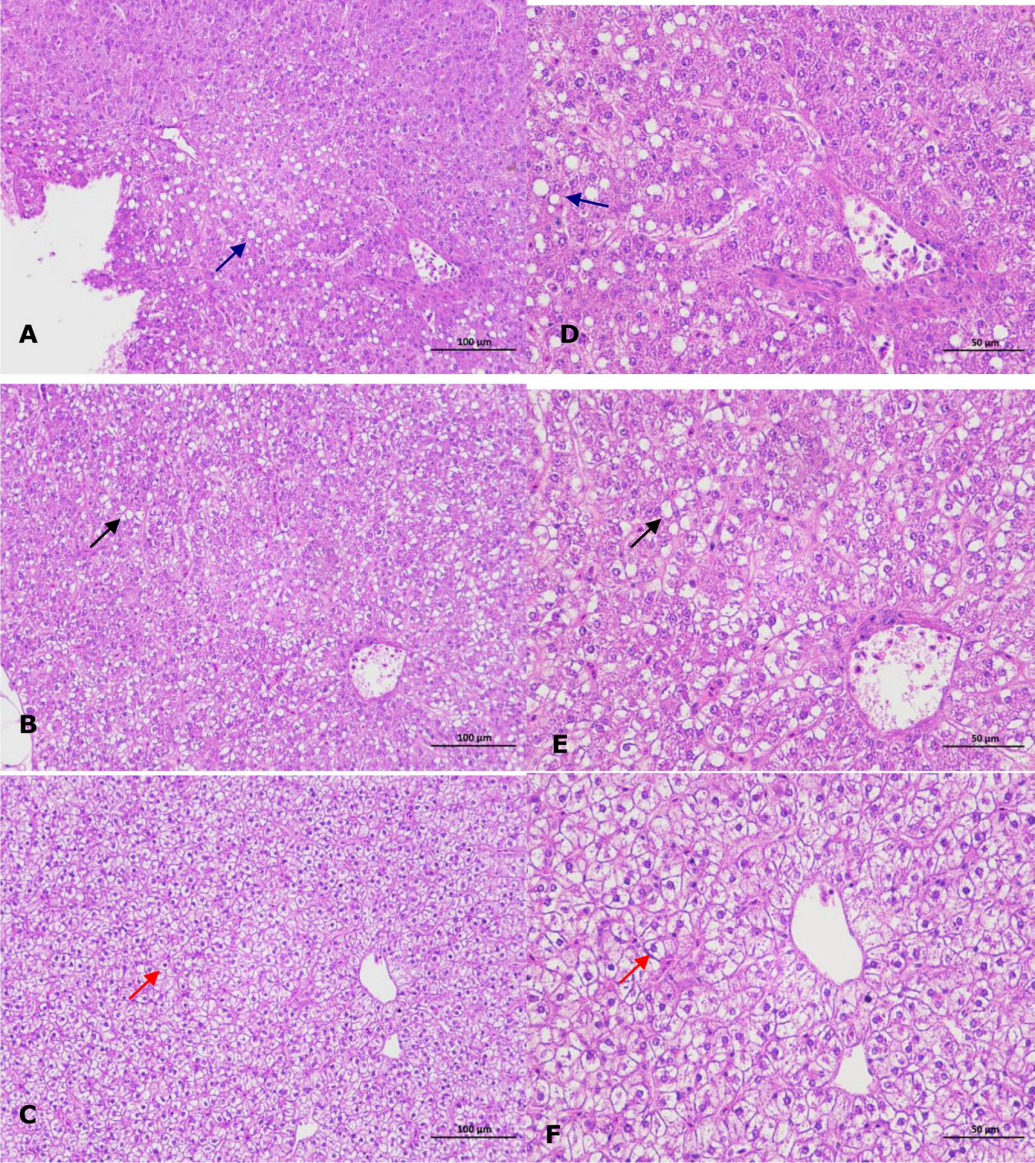
Histopathological changes in the liver of infected coral trout. **(A)** Vacuolar degeneration of liver cells (blue arrow) in the artificially infected fish (×200). **(B)** Vacuolar degeneration of liver cells (black arrow) in the naturally infected fish (×200). **(C)** Normal liver cells (red arrow) in healthy fish (×200). **(D)** Vacuolar degeneration of liver cells (blue arrow) in the artificially infected fish (×400). **(E)** Vacuolar degeneration of liver cells (black arrow) in the naturally infected fish (×400). **(F)** Normal liver cells (red arrow) in healthy fish (×400).

No parasites were detected in the naturally diseased coral trout, and no mortality or visible disease signs were observed in all tested fish challenged with the bacteria-free organ filtrate (data not shown), revealing that the disease did not result from parasites or viruses. In addition, 5 different bacterial strains, temporarily named DX1, DX2, DX3, DX4, and DX5, were isolated from the liver of naturally-diseased coral trout, and no disease signs or mortalities were noted in the control or challenged fish with isolates DX1, DX3, DX4 and DX5. Only the test fish challenged with the most dominant isolate DX2 at 3.0 × 10^7^ CFU mL^-1^ were found to exhibit a cumulative mortality of 100% (data not shown), indicating that isolate DX2 was pathogenic to the coral trout. The test fish challenged with isolate DX2 displayed the liver necrosis sign, similar to that noted in the naturally-infected fish (**Figure 1**), and the same strain (DX2), confirmed by phenotypic and molecular identification, was re-isolated from the experimental diseased fish. Furthermore, the vacuolar degeneration of liver cells was observed in the artificially and naturally infected coral trout (**Figure 2**). Yet no pathological symptoms or mortalities were observed in the control and treatment fish challenged with other bacterial isolates (data not shown). Thus, according to Koch’s postulates (Fredericks and Relman, 1996), isolate DX2 was identified as the causative pathogen of the coral trout.

### Identification of causative pathogen

**Figure 3:**
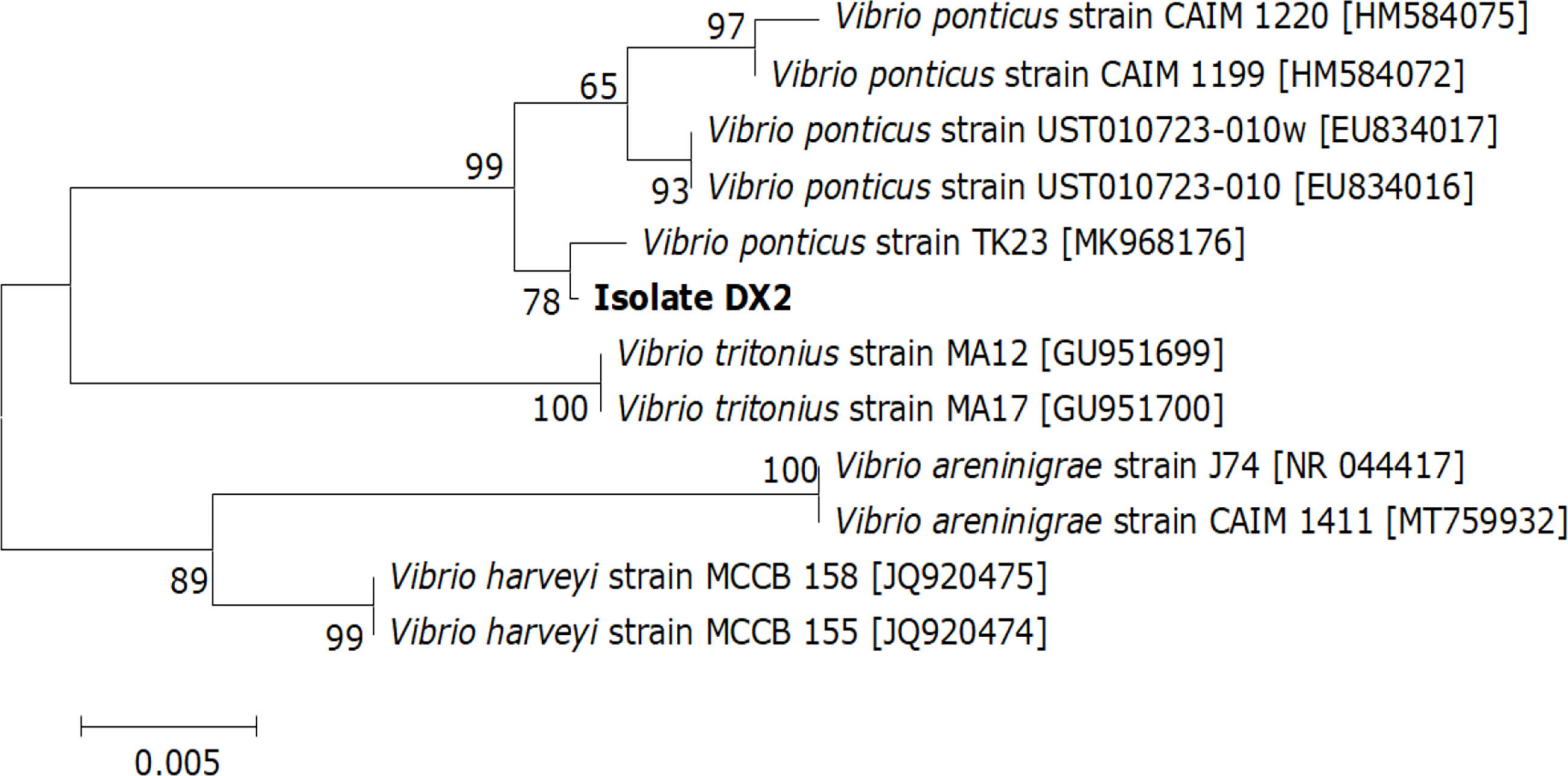
Neighbor-joining phylogenetic tree based on the 16S rRNA gene sequences of the DX2 isolate and 11 known bacteria. The GenBank accession numbers are shown beside the strain names, bootstrap values (%) are indicated beside the clades, and scale bars represent distance values.

A similarity of 99% to 100% was observed between the DX2 isolate (GenBank accession no. OP630658) and other *V. ponticus* strains in the GenBank database and further demonstrated as a *V. ponticus* strain through the phylogenetic tree (**Figure 3**). In addition, the DX2 isolate possessed identical phenotypic features to *V. ponticus* strains reported previously (Kim et al., 2007; Liu et al., 2018; You, 2018) (**Table S1**). It was positive for oxidase, β-galactosidase, lysine decarboxylase, and indole, and could utilize mannitol and sucrose, but was negative for arginine dihydrolase, ornithine decarboxylase, tryptophan deaminase, and Voges-Proskauer reaction, and could not utilize adonitol, amygdalin, arabinose, citrate, gelatine, glucose, inositol, melibiose, rhamnose, sodium thiosulfate, sorbitol, and urea. Thus, the DX2 isolate was identified molecularly and phenotypically as *V. ponticus.*

### Virulence of causative pathogen

**Figure 4:**
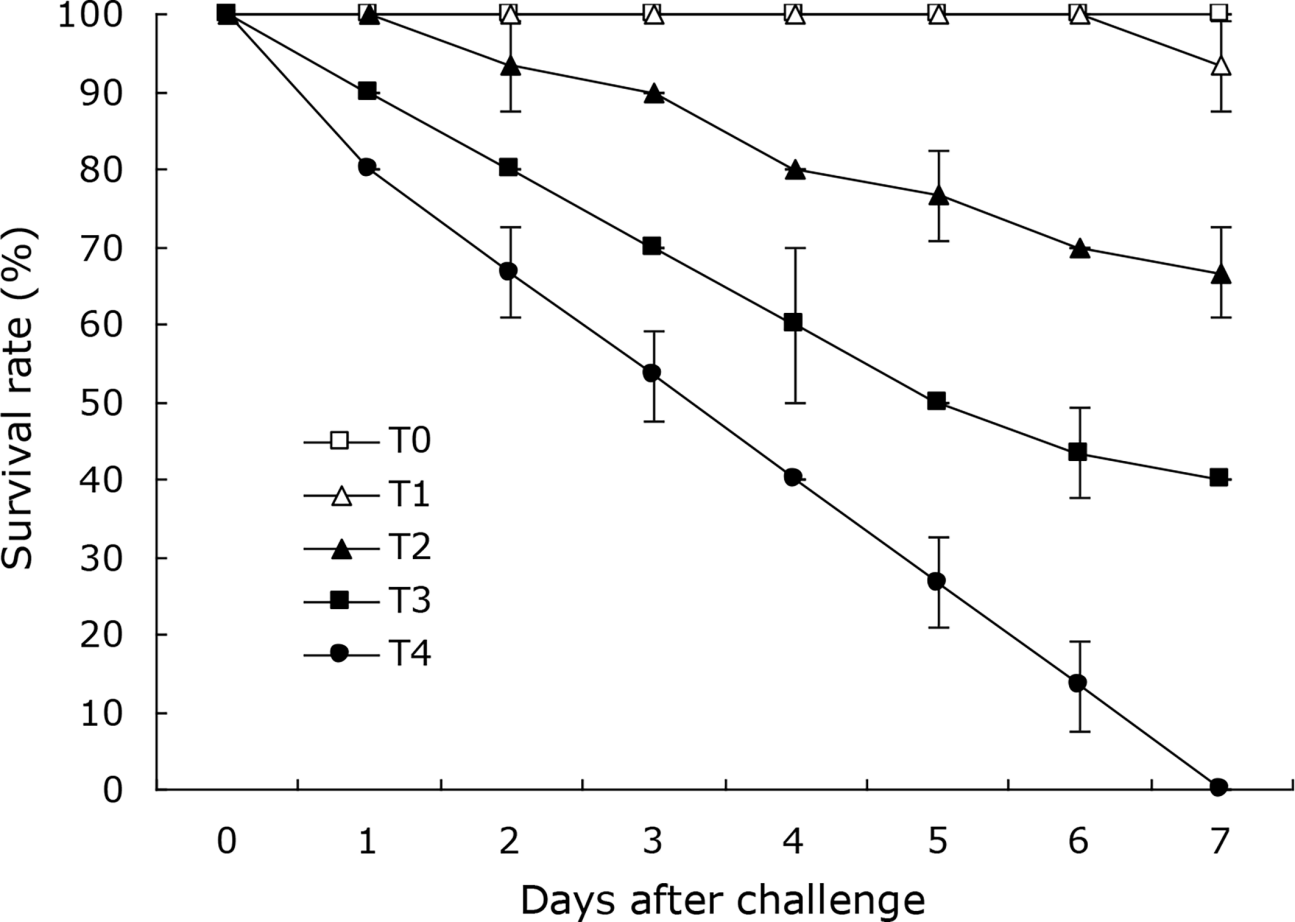
Survival rates of experimental coral trout infected by the DX2 isolate. T0, 0 CFU mL^-1^; T1, 2.1 × 10^4^ CFU mL^-1^; T2, 2.1 × 10^5^ CFU mL^-1;^ T3, 2.1 × 10^6^ CFU mL^-1;^ T4, 2.1 × 10^7^ CFU mL^-1^. Data are presented as mean ± standard deviation.

Cumulative mortalities of 6.7%, 33.3%, 60.0% and 100.0% was respectively reached in the coral trout challenged with the DX2 isolate at the cell densities of 2.1 × 10^4^, 2.1 × 10^5^, 2.1 × 10^6^, and 2.1 × 10^7^ CFU mL^-1^ (**Figure 4**) during the 7-day challenge, which showed the typical disease sign of liver necrosis. No mortality was observed in the control coral trout. Furthermore, the DX2 isolate was re-isolated from the experimental diseased fish, which was confirmed through phenotypic and molecular identification. These findings indicated that the LD_50_ value for the DX2 isolate was 6.64×10^5^ CFU mL^-1^ in the coral trout.

### Antibiotic susceptibility of causative pathogen

The DX2 isolate was highly susceptible to chloramphenicol, doxycycline, enoxacin, florfenicol, netilmicin, nalidixic acid, pipemidic acid, and polymyxin B, intermediately susceptible to gentamycin, kanamycin, novobiocin, and rifampicin, and showed multiple resistances to amoxicillin, ampicillin, azithromycin, bacitracin, cefotaxime, cefradine, ceftizoxime, cotrimoxazole, erythromycin, kitasamycin, oxacillin, penicillin, and roxithromycinum (**Table S2**). These findings indicated that the phenicols and tetracyclines antimicrobials in aquaculture use such as doxycycline and florfenicol could be chosen for the control of isolate DX2.

## Discussion

To date, several bacterial pathogens such as *Listonella anguillarum*, *Photobacterium damsela*, and *Vibrio harveyi* have posed potential risks to the coral trout aquaculture (Xu et al., 2014; Yao et al., 2015; Xu et al., 2019), which have caused a high reduction in the coral trout production. However, *V. ponticus* infection in the coral trout is scarcely documented. Previous studies have indicated that the fish liver is the primary target organ in bacterial infections (Oh et al., 2019; Malick et al., 2020). Thus, in this study, we isolated bacteria from the liver of diseased coral trout, and further demonstrated *V. ponticus* DX2 as a causative agent of coral trout, and described its phenotypic characterization. To our knowledge, this is the first report of *V. ponticus* pathogenic to coral trout.

The liver is a vital organ that can regulate the immune response in fish when exposed to bacterial infections (Dawood, 2021), and the liver necrosis is usually found in the typical signs of vibriosis in the coral trout (Xu et al., 2014). In this study, the coral trout challenged with *V. ponticus* DX2 exhibited typical liver necrosis with cell vacuolar degeneration. Similar pathological changes were also observed in the freshwater catfish infected by *Vibrio mimicus* (Geng et al., 2014). This is probably attributed to the release of bacterial toxins that causes severe liver damage (Huizinga et al., 1979). Besides, the DX2 isolate in this study showed an LD_50_ value of 6.64×10^5^ CFU mL^−1^ in the coral trout, and was classified as a strong virulent strain according to the degree of virulence described by Mittal et al. (1980). This implies that the pathogenic *V. ponticus* could probably present a threat to the health of coral trout. Surely, other primary factors were also believed to contribute to this disease, such as environmental mismanagement, and poor feed quality (Di et al., 2019; Wen et al., 2019).

Multiple antibiotic resistance in bacterial pathogens has emerged as an issue of global concern because of the dissemination of antibiotic resistance plasmids (Choudhury et al., 2012). Pathogenic isolates of *V. ponticus* in fish have been found to develop multiple resistances to macrolides and penicillins antimicrobials. For example, *V. ponticus* KJS1 in maroon clownfish was resistant to erythromycin and oxacillin (Kim et al., 2007). *V. ponticus* Lc-2013-G1 in large yellow croaker was resistant to azithromycin, erythromycin, oxacillin, and penicillin (You, 2018). In our study, the same antimicrobial resistance against macrolides and penicillins antimicrobials was also found in *V. ponticus* DX2, which showed multiple resistances against cephalosporins, peptides and sulfonamides drugs. Thus, more attention should be given to the control of fish-pathogenic *V. ponticus*.

Doxycycline and florfenicol are most commonly used veterinary antibiotics in China (Qian et al., 2021). Previous studies have demonstrated the clinical safety of oral treatment with doxycycline and florfenicol in fish aquaculture (Gaunt et al., 2013; Oliveira et al., 2022), and oral administration of doxycycline and florfenicol are effective in controlling mortality from enteric septicemia of channel catfish *Ictalurus punctatus* and edwardsiellasis of yellow catfish *Pelteobagrus fulvidraco* (Gaunt et al., 2013; Xu et al., 2021). In the present study, the DX2 isolate was highly susceptible to doxycycline and florfenicol. This serves as a reminder that doxycycline and florfenicol can be used to treatment *V. ponticus* infection in the coral trout.

## Conclusion

In this study, a virulent bacterial strain (DX2) was isolated from diseased coral trout suffering liver necrosis with cell vacuolar degeneration and was identified molecularly and phenotypically as *V. ponticus*. The findings of this study for the first time identified *V. ponticus* DX2 as a causative pathogen of diseased coral trout, and provided insights into the control of *V. ponticus* in the coral trout.

## Data availability statement

The datasets presented in this study can be found in online repositories. The names of the repository/repositories and accession number(s) can be found in the article/**Supplementary Material**.

## Ethics statement

The animal study was reviewed and approved by Institutional Animal Ethics Committee of Shanghai Ocean University.

## Author contributions

CG and JL performed the experiments and wrote the manuscript. XZ collected samples and made a formal analysis. LX and HY reviewed and edited the manuscript. All authors contributed to the article and approved the submitted version.
