# Supplementary material for: Identification of Vibrio ponticus as a bacterial pathogen of coral trout Plectropomus leopardus
Source: Front Cell Infect Microbiol. 2022 Dec 23;12:1089247. doi: 10.3389/fcimb.2022.1089247 (PMC9816427; doi:10.3389/fcimb.2022.1089247)
Supplement: Supplementary file 1 [file Table_1.doc]

**TABLE S1** Phenotypic characterization of isolate DX2.

| **Characteristics** | **Reaction** | |
| --- | --- | --- |
|  | **DX2** | ***V. ponticus*^a^** |
| [Arginine](app:ds:suberate) dihydrolase | R^-^ | R^-^ |
| Oxidase | R^+^ | R^+^ |
| [β](app:ds:glycogen)-Galactosidase | R^+^ | R^+^ |
| Gelatine | R^-^ | R^-^ |
| Lysine decarboxylase | R^+^ | R^+^ |
| [Ornithine](app:ds:malonate) decarboxylase | R^-^ | R^-^ |
| Tryptophan deaminase | R^-^ | ND |
| [Urea](app:ds:sucrose) | R^-^ | R^-^ |
| Citrate | R^-^ | R^-^ |
| Voges-Proskauer | R^-^ | R^-^ |
| Adonitol | R^-^ | R^-^ |
| Indole | R^+^ | R^+^ |
| Sodium thiosulfate | R^-^ | R^-^ |
| Arabinose | R^-^ | R^-^ |
| Amygdalin | R^-^ | R^-^ |
| Glucose | R^-^ | R^-^ |
| Inositol | R^-^ | R^-^ |
| Mannitol | R^+^ | R^+^ |
| Melibiose | R^-^ | ND |
| Rhamnose | R^-^ | R^-^ |
| Sucrose | R^+^ | R^+^ |
| [Sorbitol](app:ds:histidine) | R^-^ | R^-^ |

R^+^: positive reaction; R^-^: negative reaction; ND: not detected. a: data previously reported (Kim et al., 2007; Liu et al., 2018; You, 2018).
