# Supplementary material for: Identification of Vibrio ponticus as a bacterial pathogen of coral trout Plectropomus leopardus
Source: Front Cell Infect Microbiol. 2022 Dec 23;12:1089247. doi: 10.3389/fcimb.2022.1089247 (PMC9816427; doi:10.3389/fcimb.2022.1089247)
Supplement: Supplementary file 2 [file Table_2.doc]

**TABLE S2 Susceptibility of isolate DX2 to antibiotics.**

| **Antibiotics** | **Content**  **(μg/disc)** | **Inhibition zone diameter (mm)** | **Susceptibility** | |
| --- | --- | --- | --- | --- |
|  |  |  | **Isolate DX2** | ***V. ponticus*^a^** |
| Amoxicillin | 20 | 0±0 | R | ND |
| Ampicillin | 10 | 0±0 | R | R |
| Azithromycin | 15 | 0±0 | R | R |
| Bacitracin | 10U | 0±0 | R | R |
| Cefotaxime | 30 | 0±0 | R | R |
| Cefradine | 30 | 0±0 | R | R |
| Ceftizoxime | 30 | 0±0 | R | R |
| Cotrimoxazole^*^ | 1.25/23.75 | 0±0 | R | R |
| Chloramphenicol | 30 | 27.33±2.52 | S | S |
| Doxycycline^*^ | 30 | 20.67±0.58 | S | S |
| Enoxacin | 10 | 24.53±0.45 | S | ND |
| Erythromycin | 15 | 0±0 | R | R |
| Florfenicol^*^ | 30 | 29.33±1.15 | S | S |
| Gentamycin | 10 | 13.70±0.26 | I | I |
| Kanamycin | 30 | 15.67±1.15 | I | I |
| Kitasamycin | 15 | 0±0 | R | ND |
| Netilmicin | 30 | 21.67±0.58 | S | ND |
| Nalidixic acid | 30 | 28.67±2.31 | S | S |
| Novobiocin | 30 | 13.33±1.53 | I | ND |
| Oxacillin | 1 | 0±0 | R | R |
| Penicillin | 10U | 0±0 | R | R |
| Pipemidic acid | 30 | 25.33±0.58 | S | ND |
| Polymyxin B | 300 | 13.17±1.26 | S | ND |
| Rifampicin | 5 | 15.67±2.08 | I | I |
| Roxithromycinum | 15 | 0±0 | R | ND |
| Tobramycin | 10 | 15.50±0.56 | S | ND |

Data are presented as mean ± standard deviation.

a: the antibiotic susceptibility for *V. ponticus* was reported by Kim et al. (2007), You (2018), Liu et al. (2018) and Kumari et al. (2020).

S: highly susceptible; I: intermediately susceptible; R: resistant. ND: not detected. ^*^Antibiotics in aquaculture use.
